# Prophylactic bilateral central neck dissection should be evaluated based on prospective randomized study of 581 PTC patients

**DOI:** 10.1186/s12902-021-00909-0

**Published:** 2022-01-04

**Authors:** Shouyi Yan, Jiafan Yu, Wenxin Zhao, Bo Wang, Liyong Zhang

**Affiliations:** 1 Department of Thyroid and Vascular Surgery, Fujian Medical University Union Hospital, Fuzhou, 350001 Fujian Province China; 2 Department of General Surgery, Fujian Medical University Union Hospital, Fuzhou, 350001 Fujian Province China; 3 Minimal Invasive Center, Fujian Medical University Union Hospital, Fuzhou, 350001 Fujian Province China; 4 Fujian Medical University, Fuzhou, 350108 Fujian Province China

**Keywords:** Parathyroid protection, Papillary thyroid cancer, Central lymph node dissection, Thyroidectomy, Tumor recurrence

## Abstract

**Background:**

Prophylactic central lymph node dissection (PCND) was a basic consensus for patients with papillary thyroid carcinoma (PTC) in China. However, unilateral or bilateral central lymph node dissection (CND) was still controversial. This study aimed at investigating the safety and long-term benefit for the patients undergone with bilateral central lymph node dissection (BCCD).

**Methods:**

581 patients were enrolled and divided randomly into the test and control groups according to range of CND. 285 patients were prospectively assigned to undergo thyroid lobectomy plus BCND in the test group, other 296 patients were assigned to undergo thyroid lobectomy plus ipsilateral central lymph node dissection (ICND) in the control group.

**Results:**

We found that the numbers of total LN and pN1a in the test group were more than that of the control group (*p* = 0.002,0.004), but there was no difference in the number of metastasized lymph nodes (*p* = 0.857) and tumor recurrence (*p* = 0.308). Additionally, in the aspect of postoperative complication (1 day after surgery), the serum levels of parathyroid hormone in the BCND group were lower than that in the ICND group (*P* = 0.010), and the numbers of transient laryngeal nerve palsy were more than that(*p* = 0.033). Meanwhile, we further found that pathological tumor size larger than 1 cm and tumor side lymph node metastasis were independent risk factors for contralateral central lymph node metastasis(*p* = 0.002,0.001).

**Conclusion:**

BCND may be an alternative for patients with tumor sizes larger than 1 cm, but it would significantly increase the rate of transient vocal cord palsy, parathyroid auto transplantation and decreased PTH, but the risk of permanent complications was similar to the ICND group.

## Background

Central lymph node involvement was common in patients with papillary thyroid carcinoma (PTC) [1], which may affect tumor recurrence and patient’s survival [2–4], but PCND had not been recommended by American thyroid association (ATA) guidelines because it could obviously increase surgical complication which were caused by injury of the parathyroid gland (PG) and recurrent laryngeal nerve (RLN). Nowadays with the improvement of RLN and PG protection, the safety of CND has been confirmed based on the existing study [5], and PCND was also recommended by our guideline in China [6]. Even central lymph node metastasis did not affect the survival rate of patients, once occurred, it would increase outpatient number, cost, and the psychological pressure especially.

When confirmed with PTC, thyroid lobectomy plus PCND were considered as the first option for the patient with unilateral (cN0) PTC in china [6], and occult lymph node metastases were also found frequently in the final paraffin pathology, so there was no debate in the role of the PCND in China [7, 8], except for the range of the PCND. Basing on the safety of the ipsilateral PCND, bilateral PCND including removal of prelaryngeal, pretracheal, and bilateral paratracheal nodes was proposed in patients with unilateral PTC in recent years [9, 10].

BCND could decrease the risk of overlooking contralateral metastases but add a higher risk of RLN palsy and hypoparathyroidism. Now with the development of RLN and PG protection, the risk mentioned above had decreased significantly. More comparative studies had suggested that BCND may be an effective alternative to ICND for cN0 PTC because of its long-term oncologic outcome and the similar complications [11].

Although more retrospective studies reported that there were many occult metastasis lymph nodes in the contralateral region, there were still many controversies in the field of tumor recurrence and metastasis for lacking a prospective randomized controlled study. It was hard to sure if the patients with CN0 PTC would benefit from BCND in the long-term oncologic outcome [12, 13]. In the present study, prospective randomized controlled studies with standard BCND were rare, especially the dissecting range and operative process. We aimed to prospectively compare the results of ICND and BCND and provide clinical evidence for bilateral central lymph node dissection in patients with unilateral cN0 PTC.

## Methods

### Patients’ characteristics

A total of 581 consecutive patients were enrolled in this study from 2015.12 to 2017.12. The characteristics of patients were shown in Table 1. All the patients were diagnosed as unilateral cN0 PTC. They were randomly assigned to one of the following groups: 1. Thyroid lobectomy plus ICND (control group); 2. Thyroid lobectomy plus BCND (test group). Exclusion criteria included previous neck surgery, any preoperative (i.e., clinical and ultrasound examination) evidence of bilateral PTC, macroscopically infiltrating tumors, diagnosed with T4 classification preoperatively, isthmus cancer, age more than 55 years, or distant metastases. The randomization was done by using computer-generated random number tables. All the operations were performed by the same surgeon. The study had been registered with North America Clinical Practice (NCT02648399) and approved by the Ethics Committee of the Fujian Medical University Union Hospital (2015KY026), and all patients provided informed consent.

**Table 1:** Characteristics of all included patients are reported

Characteristics	ICND (Control group)	BCND (Test group)	*p* value
No. of patients	296	285	/
Age [years; ±SD (range)]	43.2 ± 11.7	43.8 ± 11.5	0.544
Male/female	70/226	72/213	0.939
Pathological tumor size [mm; ±SD (range)]	8.3 ± 5.0	8.8 ± 5.5	0.356
Operative time [min; ±SD (range)]	50.5 ± 10.4	60.4 ± 11.5	0.006
Pathological T classification (<T3/T3)	157/139	167/118	0.178
Pathological N classification(p N0 / p N1a)	134/162	96/189	0.004
Tumor side/contralateral side (+/+)	/	61	/
Tumor side/contralateral side (+/−)	/	124	/
Tumor side/contralateral side (−/+)	/	4	/
Tumor side/contralateral side (−/−)	/	96	/
Unifocal/Multifocal	242/54	245/40	0.169
Transient hypocalcemia	40/296	47/285	0.315
Transient laryngeal nerve palsy	13/296	25/285	0.033
Definitive laryngeal nerve palsy	2/296	2/285	1.000
Post-operation hemorrhage	6/296	5/285	1.000
Removed lymph nodes [±SD (range)]	8.5 ± 5.8	12.5 ± 6.5	0.002
Metastasized lymph nodes [±SD (range)]	2.6 ± 1.5	2.88 ± 1.7	0.857
PG numbers of auto-transplantation	226/592	377/855	0.025
The ratio of IONM	90/296	85/285	0.879
PTH (pre-operation)	4.28 ± 1.50	4.18 ± .1.62	0.945
CA (pre-operation)	2.34 + 0.16	2.32 ± 0.10	0.243
PTH (Postoperative 1 Day)	2.83 ± 1.37	2.55 ± 1.21	0.010
CA (Postoperative 1 Day)	2.26 ± 0.10	2.26 ± 0.09	0.556
PTH (Postoperative 6 months)	3.93 ± 1.93	3.95 ± 1.99	0.915
CA (Postoperative 6 months)	2.37 ± 0.93	2.36 ± 0.95	0.949
Number of tumor recurrence	0/296	1/285	0.491

### Study endpoints

The primary endpoint was to compare the long-term (2 years) oncological outcome (it refers to tumor recurrence which was found in the original surgical area, the contralateral thyroid lobe or the lateral neck in this study. And the patient did not receive further treatment, such as completion thyroidectomy or radioactive iodine, before the recurrence was detected), while the secondary aim of the study was to compare postoperative complications, operative time, and the numbers of total and metastatic LN, pathological T and N classification, and pathological tumor size between the two groups.

### Definitions

**Fig. 1:**
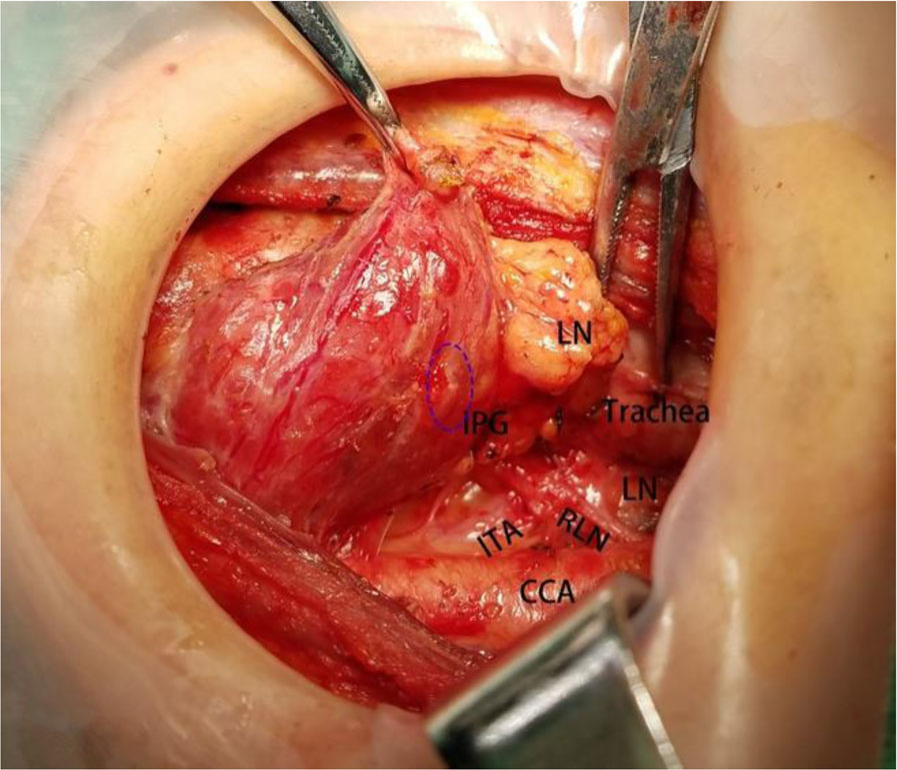
After separating the outer side of the thyroid gland and pulling the lower pole of the thyroid lobe upward and inside, then we could quickly find the RLN and protect the PG.

**Fig. 2:**
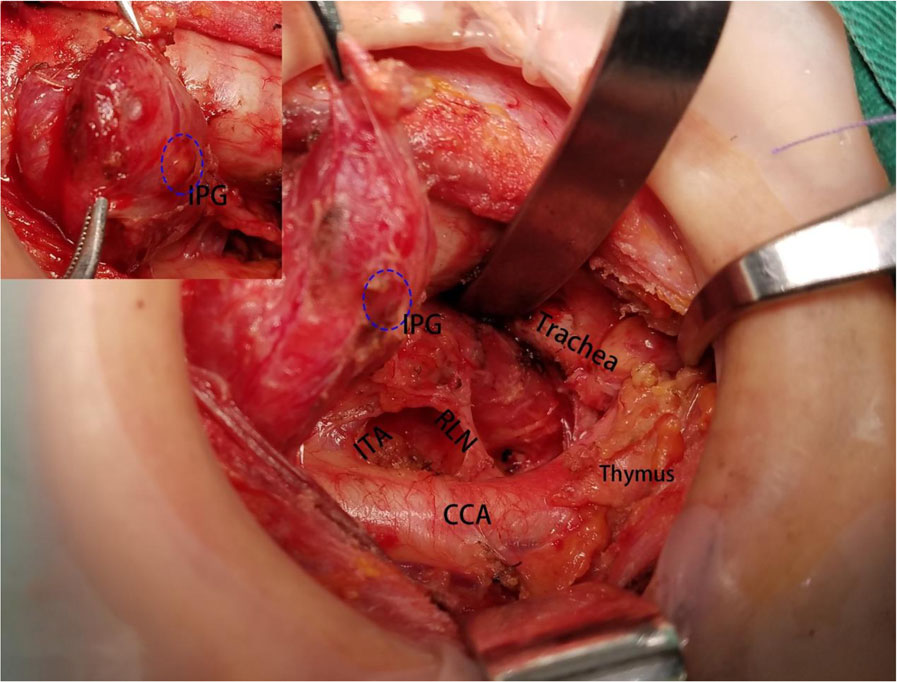
This is the anatomical display after bilateral central lymph node dissection (BCND). We can protect the recurrent laryngeal nerve and parathyroid gland well

**Fig. 3:**
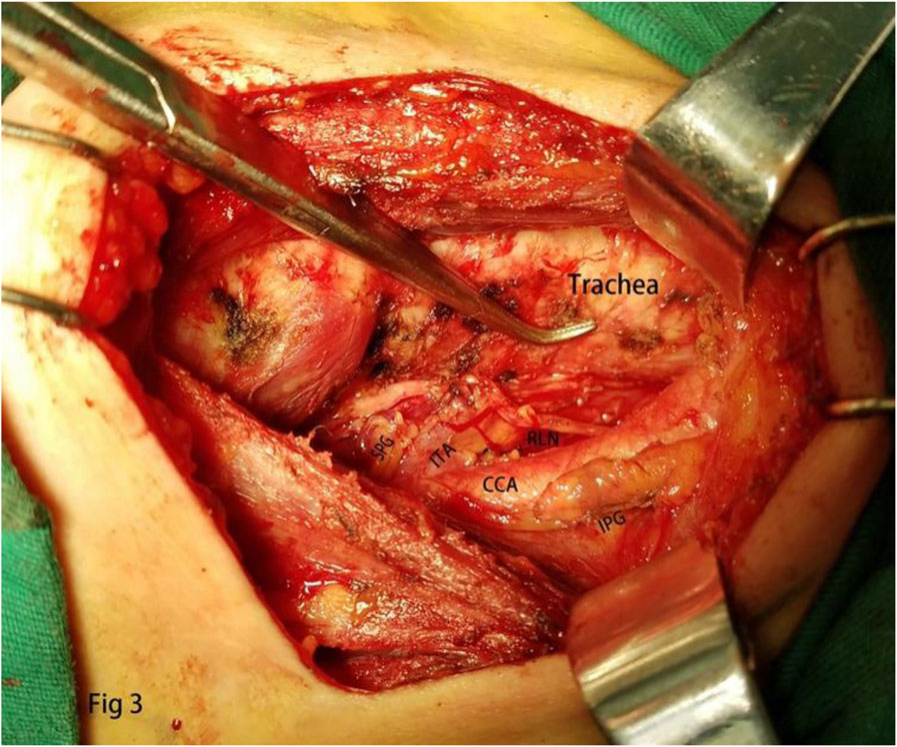
This is the anatomical display after ipsilateral central lymph node dissection (ICND)

PTC were defined as clinically cN0 in the absence of any preoperative (i.e., clinical and ultrasound examination) evidence of multifocal disease or lymph node involvement, respectively. All surgical procedures were performed by the same experienced endocrine surgeon. ICND group should remove prelaryngeal, pretracheal, and paratracheal lymph node on the tumor side. BCND group should remove prelaryngeal, pretracheal, tumor side and contralateral paratracheal lymph node. Due to the presence of the thyroid lobe, there were some differences in the dissection of the paratracheal lymph node on the contralateral side. The upper bound of the contralateral side dissection was generally located at the level of the inferior thyroid artery. First, we separated the outer side of the thyroid gland, pulled the lower pole of the thyroid lobe upward and inside, then continued separating downward to the thymus level in the caudal direction and protecting thymus vein (Fig. 1). We could find the inferior parathyroid gland, which was closely related to the thymus gland named thymus-related inferior parathyroid gland and attempt to preserve it in situ. We also find the characteristics that the right RLN intersected with the common carotid artery on the right side, but the RLN was close to the surface of the esophagus on the left side [14]. Therefore, we could quickly find the RLN in the lower part of the neck and separated along the surface of RLN to the level of the inferior thyroid artery (Fig. 2). The range of dissection under the inferior thyroid artery was consistent with that of conventional dissection of the tumor side (Fig. 3) [15]. Pathological staging was defined in accordance with the 2017 National Comprehensive Cancer Network pTNM staging system.

### **Follow-up and postoperative treatment of** hypocalcemia

Complications were considered definitive if they lasted more than six months. Laryngoscopy was performed both preoperatively and postoperatively in all patients to check vocal cord motility. Hypocalcemia was defined as blood calcium level was below the lower limit of the reference value, with or without symptoms. Calcium supplementation was not routinely administered to patients, but calcium and vitamin D were routinely prescribed to patients with symptomatic hypoparathyroidism until the level of PTH and blood calcium recovered. Intravenous substitution of calcium was not a routine unless serious symptomatic hypocalcemia was present. Postoperative suppressive levothyroxine treatment was administered to all patients. All patients underwent thyroid (parathyroid) function (TSH, T3, T4, PTH, Ca) measurements under suppressive levothyroxine treatment after 2 months of surgery and ultrasound neck scan after 12 months of surgery. Follow-up data were obtained by outpatient consultations or telephone contact.

### Data collection

General characteristics, intraoperative factors, pathologic examination, the number of lymph nodes and metastatic lymph nodes in the resected specimens, and postoperative complications were collected retrospectively. The seventh edition of the American Joint Committee on Cancer (AJCC) staging was used for all the recruited patients. The primary endpoints were tumor recurrence or metastasis, the second endpoints were the operative time, the numbers of the total LN and the metastatic LN, the ratio of PG auto-transplantation, the serum levels of PTH, the postoperative complications, the ratio of IONM.

### Statistical analysis

Statistical analysis was performed by SPSS 26.0, Chicago, IL, USA. All values were presented as mean ± standard deviation. A T-test or Chi-Square test was used to determine statistical significance, requiring *p* < 0.05 was statistically significant. And the normality of these variables should be confirmed before using t-test.

## Results

### Patents characteristics

There were 296 and 285 patients respectively in the ICND and BCND group. And no significant difference was found between the two groups concerning age, sex, pathological tumor size, number of lesions, pathological T classification, the ratio of IONM, PTH and Ca (pre-operation) (Table 1).

### Short-term results

Patients in the BCND group experienced more operative time (*p* = 0.006) and more LN numbers. (*p* = 0.004). Also, with more PG numbers of auto-transplantation and removed lymph nodes in the BCND group than that of the ICND group. But there was no significant difference in the number of metastasized lymph nodes, Pathological T classification. Additionally, in the aspect of postoperative complication, the serum levels of PTH (1 day after surgery) in the BCND group (*P* = 0.010) were lower than that in the ICND group, and the numbers of transient laryngeal nerve palsy (*p* = 0.033) were higher than that. While in the aspect of post-operation hemorrhage, Ca (1 day after surgery), the serum levels of PTH (6 months after surgery), Ca (6 months after surgery), there were no significant difference. (Table 1).

### Long-term results

No significant difference was found in the number of tumor recurrence. Additionally, in the aspect of postoperative complication, no significant difference was found including definitive hypoparathyroidism, definitive laryngeal nerve palsy. There were 65 and 220 patients, respectively, with contralateral side LN (+) and contralateral side LN (−) in the BCND group. No significant difference was found between the two subgroups in the BCND group including age, sex, pathological T classification, number of lesions, lesion located in the lower pole (Table 2), except the tumor side LN (+) (*p* = 0.000) and pathological tumor size (p = 0.000) (Table 2). Meanwhile multivariate logistic regression analysis showed that pathological tumor size(≥10 mm)(OR = 3.337) and tumor side LN (+) (OR = 3.774) were independent risk factors for contralateral central lymph node metastasis (Table 3).

**Table 2:** Risk factors for unilateral PTC with contralateral central lymph node metastasis

Characteristics	contralateral side LN(+)	contralateral side LN(−)	*p* value
No. of patients	65	220	/
age	42.4 ± 10.5	43.4 ± 11.9	0.554
Male/female	16/49	50/170	0.751
Pathological T classification (<T3/T3)	43/22	126/94	0.200
Pathological tumor size(<10/≥10 mm)	25/40	147/73	0.000
Unifocal/Multifocal	52/13	180/40	0.741
lesion located in the lower pole	31/34	114/106	0.559
Tumor side LN (+/−)	61/4	124/96	0.000

**Table 3:** Multi-variable analysis for unilateral PTC with contralateral central lymph node

variable	B	Standard error	Wald	df	Significance	OR value	95.0% C.I.for EXP(B)	
							The lower limit	The upper limit
Male/female	0.434	0.509	0.637	1	0.436	1.527	0.535	4.211
Pathological T classification (<T3/T3)	0.865	0.538	2.588	1	0.112	2.413	0.816	6.925
lesion located in the lower pole	0.502	0.566	0.692	1	0.521	1.622	0.513	4.252
Unifocal/Multifocal	0.425	0.478	0.611	1	0.476	1.498	0.520	4.328
Pathological tumor size (<10/≥10 mm)	1.411	0.476	7.302	1	0.002	3.337	1.502	9.877
Tumor side LN (+/−)	1.334	0.589	4.523	1	0.001	3.774	1.109	10.882

## Discussion

The importance of the ICND had been accepted by the most surgeons in China for the reason that occult or microscopic lymph node metastases of the tumor side were found in 50–80% of patients with CN0 PTC [6, 16, 17]. Unfortunately, ICND still carried the risk of contra central lymph node metastases being overlooked in approximately one-quarter of patients [18]. This could imply a potential risk of tumor recurrence and residual. The central lymph node contains prelaryngeal, pretracheal, bilateral paratracheal lymph node, so some scholars suggest that we should dissect bilateral central lymph node called BCND which may change the TNM stage and reduce the risks of tumor recurrence [11, 19]. Conversely, main reasons against BCND for the higher risk of complications including parathyroid and RLN injury, and there was no long-term benefit for the patients [20–22].

To dispute the controversy, we designed this study to investigate the feasibility of BCND and its impact on complications and long-term benefits. Regarding the result, BCND was associated with prolonged operative time compared with the ICND, but it just took about 10 min longer at our institution. And we found there were 54.73 and 66.32% lymph node metastases in the group of ICND and BCND respectively, it was consistent with the existing research conclusions that occult lymph node metastases are frequent in cN0 PTC, especially in the BCND group(*p* = 0.004). But there was no difference in the number of tumor recurrence, metastatic LN, and pathological T classification. Meanwhile the serum levels of PTH (1 day after surgery) in the BCND group (1 day after surgery) (*P* = 0.010) was lower than that in the ICND group, and the numbers of PG auto-transplantation (*p* = 0.025) and transient laryngeal nerve palsy (*p* = 0.033) were higher than that. Basing the result mentioned above, BCND didn’t seem to gain more benefits for the patients with cN0 PTC.

But the permanency complications rate, including the injury of permanent parathyroid and RLN, were not significantly different between the two groups. So, we seem to see a reason that BCND might be carried out. What caused it? The reasons for this were as following. First, with the help of new ways of finding RLN [14], we can protect it well, even though there were only about 30% patients with IONM because of health policy. Meanwhile, there were more patients with transient laryngeal nerve palsy in the BCND group than that of the ICND group, but all the patients recovered after 2 months in the function of the vocal cords which was confirmed by the electronic laryngoscope. Moreover, with the improvement of PG recognition and auto-transplantation, the function of the PG (Postoperative 6 months) in the BCND group was the same with groups of ICND. We had to emphasize the importance of PG transplantation as soon as possible, especially in the patents that the PG was not in situ or mis-resection [22]. With the help of new ways of finding RLN, PG recognition and auto-transplantation [23–25], BCND may be safe in the patients with cN0 PTC in our study based on the long-term results because it may provide an adequate surgical resection in patients with occult nodal disease and reduced the risk of tumor recurrence theoretically. So BCND may be a feasible treatment option, but who was suitable for BCND will be a question to be discussed.

We further found that pathological tumors size larger than 1 cm and tumor side lymph node metastasis were independent risk factors for contralateral central lymph node metastasis in patients with PTC. As the Table 2 shows, the total number of lymph nodes dissected was more than 10, so it was too difficult to make a rapid and accurate diagnosis in 30 min for the pathologist, especially in the condition of occult metastasis or large specimen, or in some primary hospitals. So, it’s not feasible that we dissect the contralateral side LN after knowing the result of the frozen pathology of the tumor side LN during operation [26]. In addition, there was no difference in tumor recurrence between the two groups that may be related to the inert biological behavior of thyroid cancer, and its follow-up time should be longer. Therefore, we do not recommend routine BCND in all, except for patients with tumor size larger than 1 cm.

The major advantage of the present study was randomization. It was clear that this method of patient selection may decrease some concerns regarding recruitment bias; meanwhile, patients in the two groups were operated during the same period, and all the involved surgeons performed both surgical procedures. But we had to point out that our study had some limitations. Firstly, the major disadvantage was the lack of follow-up data for 5/10-years tumor recurrence, even if there was no significant difference in terms of 2 -years tumor recurrence between two groups. Secondly, due to the dissection of contralateral lymph node and the destruction of the original tissue structure, it was not conducive to the next operation, and contralateral central lymph node dissection could affect the PG, therefore the status of the PG should be recorded clearly on the medical documents which was useful for next operation. Thirdly, we did not detect tumor genetic mutation and use IONM routinely.

## Conclusion

BCND may be an alternative for patients with tumor sizes larger than 1 cm, but it would significantly increase the rate of transient vocal cord palsy, parathyroid auto transplantation and decreased PTH, but the risk of permanent complications was like ICND group. And one-quarter of patients had occult nodal metastasis in the contralateral side, but long-term oncological outcome was also needed to further study the merit of BCND performed with lobectomy.

## Data Availability

The datasets during and/or analyzed during the current study available from the corresponding author on reasonable request.

## Abbreviations

PTC: papillary thyroid carcinoma
TC: thyroid carcinoma
CND: central lymph node dissection
PCND: prophylactic central lymph node dissection
ICND: ipsilateral central lymph node dissection
BCCD: bilateral central lymph node dissection
RLN: recurrent laryngeal nerve
PG: parathyroid gland
PTH: parathyroid hormone
IONM: intraoperative neuromonitoring

## References

[1] Yan S, Zhao W, Wang B, Zhang L (2018). Standardization of simple auxiliary method beneficial to total endoscopic thyroidectomy on patients with PTC, based on retrospective study of 356 cases [J]. Endocrine.

[2] Haugen BR, Alexander EK, Bible KC, Doherty GM, Mandel SJ, Nikiforov YE, Pacini F, Randolph GW, Sawka AM, Schlumberger M, Schuff KG, Sherman SI, Sosa JA, Steward DL, Tuttle RM, Wartofsky L (2016). 2015 American Thyroid Association management guidelines for adult patients with thyroid nodules and differentiated thyroid Cancer: the American Thyroid Association guidelines task force on thyroid nodules and differentiated thyroid Cancer. Thyroid.

[3] Liu FH, Kuo SF, Hsueh C (2015). Postoperative recurrence of papillary thyroid carcinoma with lymph node metastasis. J Surg Oncol.

[4] Zheng CM, Ji YB, Song CM, Ge MH, Tae K (2018). Number of metastatic lymph nodes and ratio of metastatic lymph nodes to Total number of retrieved lymph nodes are risk factors for recurrence in patients with clinically node negative papillary thyroid carcinoma. Clin Exp Otorhinolaryngol.

[5] Chereau N, Zambeli-Ljepović A, Oyekunle TO (2019). Predicting recurrence of papillary thyroid cancer using the eighth edition of the AJCC/UICC staging system. Br J Surg.

[6] Chinese society of endocrinology, Chinese society of surgery, endocrinology group, head and neck tumor committee (2013). Guidelines for the diagnosis and treatment of thyroid nodules and differentiated thyroid cancer [J]. Chin J nuclear medicine and molecular imaging.

[7] Liang J, Li Z, Fang F, Yu T, Li S (2017). Is prophylactic central neck dissection necessary for cN0 differentiated thyroid cancer patients at initial treatment? A meta-analysis of the literature. La dissezione linfonodale profilattica del compartimento centrale del collo è necessaria come trattamento iniziale nei pazienti affetti da carcinoma differenziato della tiroide cN0? Meta-analisi della letteratura. Acta Otorhinolaryngol Ital.

[8] Hennessy M, Goldenberg D (2016). The role of prophylactic central neck dissection in the treatment of differentiated thyroid Cancer. Rambam Maimonides Med J.

[9] Chen Q, Zou XH, Wei T, Huang QS, Sun YH, Zhu JQ (2015). Prediction of ipsilateral and contralateral central lymph node metastasis in unilateral papillary thyroid carcinoma: a retrospective study. Gland Surg.

[10] Holostenco V, Khafif A (2014). The upper limits of central neck dissection [J]. Jama Otolaryngology–head Neck Surg.

[11] Moo TA, Umunna B, Kato M (2009). Ipsilateral versus bilateral central neck lymph node dissection in papillary thyroid carcinoma [J]. Ann Surg.

[12] Hall CM, Snyder SK, Maldonado YM, Lairmore TC (2016). Routine central lymph node dissection with total thyroidectomy for papillary thyroid cancer potentially minimizes level VI recurrence [J]. Surgery.

[13] Lee HS, Park C, Kim SW, Noh WJ, Lim SJ, Chun BK, Kim BS, Hong JC, Lee KD (2016). Pathologic features of metastatic lymph nodes identified from prophylactic central neck dissection in patients with papillary thyroid carcinoma [J]. Eur Arch Oto Rhino Laryngol.

[14] Yan S, Xie C, Zhao W, Wang B, Zhang L (2020). A simple, efficient, and safe way of finding recurrent laryngeal nerve beneficial for PTC patients. Medicine (Baltimore).

[15] Cui Q, Li Z, Kong D, Wang K, Wu G (2016). A prospective cohort study of novel functional types of parathyroid glands in thyroidectomy: in situ preservation or auto-transplantation?. Medicine (Baltimore).

[16] Ito Y, Miyauchi A, Masuoka H, Fukushima M, Kihara M, Miya A (2018). Excellent prognosis of central lymph node recurrence-free survival for cN0M0 papillary thyroid carcinoma patients who underwent routine prophylactic central node dissection. World J Surg.

[17] Ito Y, Miyauchi A, Kudo T, Kihara M, Fukushima M, Miya A (2017). The effectiveness of prophylactic modified neck dissection for reducing the development of lymph node recurrence of papillary thyroid carcinoma [J]. World J Surg.

[18] Roh JL, Kim JM, Park CI (2011). Central lymph node metastasis of unilateral papillary thyroid carcinoma: patterns and factors predictive of nodal metastasis, morbidity, and recurrence [J]. Ann Surg Oncol.

[19] Chang YW, Lee HY, Kim HS, Kim HY, Lee JB, Son GS (2018). Extent of central lymph node dissection for papillary thyroid carcinoma in the isthmus. Ann Surg Treat Res.

[20] Xiang D, Xie L, Li Z, Wang P, Ye M, Zhu M (2016). Endoscopic thyroidectomy along with bilateral central neck dissection (ETBC) increases the risk of transient hypoparathyroidism for patients with thyroid carcinoma [J]. Endocrine.

[21] Calò PG, Conzo G, Raffaelli M (2016). Total thyroidectomy alone versus ipsilateral versus bilateral prophylactic central neck dissection in clinically node-negative differentiated thyroid cancer. A retrospective multicenter study. Eur J Surg Oncol.

[22] Sitges-Serra A, Lorente-Poch L, Sancho J (2018). Parathyroid autotransplantation in thyroid surgery. Langenbecks Arch Surg.

[23] Mangano A, Kim HY, Wu CW, Rausei S, Hui S, Xiaoli L, Chiang FY, Roukos DH, Lianos GD, Volpi E, Dionigi G (2016). Continuous intraoperative neuromonitoring in thyroid surgery: safety analysis of 400 consecutive electrode probe placements with standardized procedures. Head Neck.

[24] Shouyi Y, Wenxin Z, Bo W (2018). A novel Technology for Localization of parathyroid adenoma: ultrasound-guided fine needle aspiration combined with rapid parathyroid hormone detection and Nano-carbon technology. Surg Innov.

[25] Yan S, Zhao W, Wang B, Zhang L (2018). Preoperative injection of carbon nanoparticles is beneficial to the patients with thyroid papillary carcinoma: From a prospective study of 102 cases. Med (Baltimore).

[26] Raffaelli M, De Crea C, Sessa L (2015). Ipsilateral central neck dissection plus frozen section examination versus prophylactic bilateral central neck dissection in cN0 papillary thyroid carcinoma [J]. Ann Surg Oncol.

